# Supplementary material for: Transcriptome Analysis Reveals the Molecular Regularity Mechanism Underlying Stem Bulblet Formation in Oriental Lily ‘Siberia’; Functional Characterization of the LoLOB18 Gene
Source: Int J Mol Sci. 2022 Dec 3;23(23):15246. doi: 10.3390/ijms232315246 (PMC9738039; doi:10.3390/ijms232315246)
Supplement: Supplementary file 1 [file ijms-23-15246-s001.zip › ijms-2002855-supplementary.pdf]

International Journal of *Molecular Sciences*

MDPI

Transcriptome Analysis Reveals the Molecular Regularity Mechanism Underlying Stem Bulblet Formation in Oriental Lily 'Siberia'; Functional Characterization of the *LoLOB18* Gene

Shaozhong Fang [1,†], Chenglong Yang [1,†], Muhammad Moaaz Ali [2], Mi Lin [1], Shengnan Tian [2], Lijuan Zhang [2], Faxing Chen [2] and Zhimin Lin [1,*]

Table S1. Summary of sample sequencing data quality.

sample	raw_reads	clean_reads	clean_bases	error_rate	Q20	Q30	GC_pct
AI_1	23940635	23023593	6.91G	0.03	97.05	92.49	45.76
AI_2	26335661	24931990	7.48G	0.03	97.19	92.68	46.53
AI_3	23631994	22749394	6.82G	0.03	97.79	93.81	49.74
AII_1	23333055	22242572	6.67G	0.03	97.46	93.18	46.98
AII_2	22998623	22007557	6.6G	0.03	97.6	93.43	47.13
AII_3	22686930	21628791	6.49G	0.03	97.65	93.47	49.03
AIII_1	23382409	22517722	6.76G	0.03	97.55	93.25	47.63
AIII_2	23922151	22604843	6.78G	0.03	97.6	93.26	49.09
AIII_3	23025309	21890514	6.57G	0.03	97.52	93.21	48.23
BI_1	22760794	22291418	6.69G	0.02	98.55	95.48	47.45
BI_2	25263161	24507882	7.35G	0.03	97.6	93.36	49.84
BI_3	23510572	22740522	6.82G	0.03	97.63	93.42	50.67
BII_1	23099381	22342918	6.7G	0.03	97.73	93.61	50.29
BII_2	23503526	22691422	6.81G	0.03	97.63	93.41	50.27
BII_3	23393160	22605723	6.78G	0.03	97.75	93.65	50.1
BIII_1	23694202	22980909	6.89G	0.03	97.71	93.51	49.54
BIII_2	23625022	22302139	6.88G	0.03	97.89	93.76	49.58
BIII_3	23767045	23075120	6.92G	0.03	97.7	93.51	49.85

AI – vertical (control) group after 20 days of planting; AII – horizontal group after 20 days of planting; AIII – slant group after 20 days of planting; BI – vertical (control) group after 50 days of planting; BII – horizontal group after 50 days of planting; BIII – slant group after 50 days of planting.

Table S2. The primer pairs used in study.

Gene	Primer sequence（5'–3'）	Temperature (℃)	Amplification Size
LoLOB18	F:ATGAGCTGCAGTACGAGCAGCG<br>R:TCACCTAGAGACTGGAGGAGTAT	56	687 bp
TRV-LOB18	F:TGAGTAAGGTTACCGGAATTCACTATCTGCTACGAGGCACAGG<br>R:GGGACATGCCCGGGCCTCGAGGTTGTTGTTGCTGCTGGAGGTT	58	340 bp
qPCR-LOB18	F:TTCCAACTCCACCCTCCTTGTC<br>R:CTCCACCACCACTGCTGCTA	57.5	180 bp
coat protein	F:CCTGCTGACTTGATGGACGA<br>R:CCAGTGTTCGCCTTGGTAGT	55.5	359 bp
qPCR-SUS	F:TATTCTGAGAGGCTGATGACACT<br>R:CAAGTTACGGTATTTGAGGGCATA	55.2	122bp
qPCR-SUAR	F:TCTCATCTCACACCTTGCCAT<br>R:CCTCCTGCTCATCATACTTCCT	55	144bp
qPCR-GID	F:GAGAAATGAAACGGCAATCG<br>R:CTCAAATACCAGGTCCTCCC	55.8	109
qPCR-SLR1	F:GTGGTTGAACAAGAAGCAAATC<br>R:CGAACATAGTAGAGTAGTAGTGG	55.5	84bp
qPCR-XTH23	F:GACTTCCACACTTACTCCATCGT<br>R:CCATACCAGACACAGGCATCAG	56.2	256bp
qPCR-AGPS	F:GCTTCTTGCGTGAATGTGGTAT<br>R:ATCTTCTGGTCTATCGGCTTCTT	56	274bp
qPCR-GH3.8	F:GCTGATGGAGTACGCTATT<br>R:ATGCTCTAAGTGGAGAATGG	56.4	195bp
qPCR-EF	F:GGCACTAACTCGCTCCTTCTG<br>R:TTGGTAAGATGCTGGTGATTGGAT	56	173bp
qPCR-KNOX1	F:CTTCACCTCACACCTTCTACCA<br>R:GCCTTGACGGACTCCACAT	55.4	146bp

Table S3. Annotation of assembled unigenes against seven public databases.

Database	Number of Unigenes	Percentage
Annotated in NR	63762	43.81
Annotated in NT	53444	36.72
Annotated in KO	27326	18.77
Annotated in SwissProt	56595	38.88
Annotated in PFAM	54137	37.19
Annotated in GO	54133	37.19

Annotated in KOG	24441	16.79
Annotated in all Databases	13913	9.56
Annotated in at least one Database	145530	100
Total Unigenes	145530	100

Table S4. The sequences of *LoLOB18*.

ATGAGCTGCAGTACGAGCAGCGGAGCTGGCGGTGGCGCCGCCGGTGGCGGCAGTGGTAGCGGAGGCGGC
GGTCCGTGCGGGGCATGCAAGTTCCTGAGGAGGAAGTGCGTGAGCGGGTGCATTTTTGCTCCTTACTTCGA
CTCCGAGCAGGGAGCGGCGCATTTCGCCGCCGTGCACAAGGTGTTTGGTGCGAGCAATGTGTCGAAGCTG
CTGCTGCATATTCCGGCACACAAGAGGCTGGATGCGGTCATCACTATCTGCTACGAGGCACAGGCGAGGC
TGCGGGATCCTGTCTATGGATGTGTTGCTCATATATTTGCGCTGCAGCAGCAGGTGGTGAACCTACAGGCC
GAGCTCTCCTACTTACAAGCCCACCTGGCAACCCTAGAGCTACCATCTCCTCCTCCGCCGCCGCCCGCAAT
TCCAACTCCACCCTCCTTGTCCATGTCTGACCTGCCCTCTGCCTCGAACATACCCACCTACCTGGACCTCTC
CAGCCTCTTTGATCCCCCTCAGCAGCCCCCGTGGAACCTCCAGCAGCAACAACAACAGGTTCAACTCCGAC
AGACGCTTGGGTCCGGTAGCAGCAGTGGTGGTGGAGACCTCCATGCACTGGCGAGGGAACTCCTGGGTCG
CCACCGATGCCCGCCAGGCGATACACTGGATACTCCTCCAGTCTCTAGGTGA

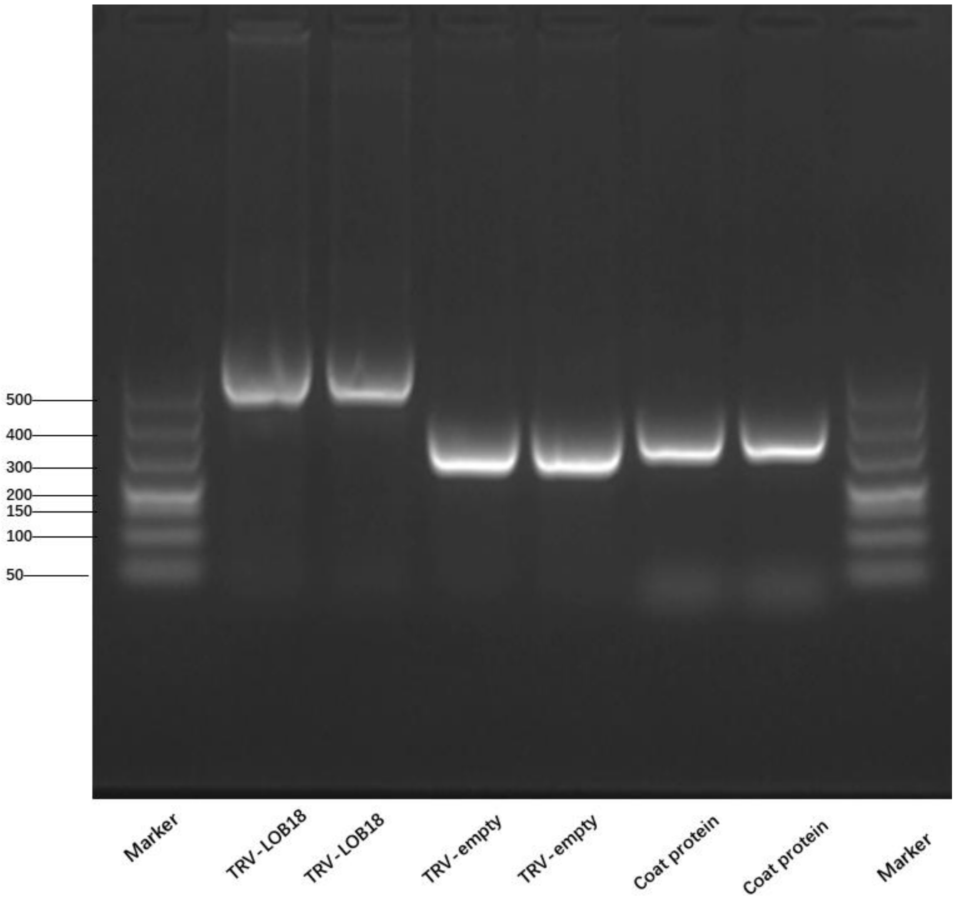

Figure S1. The figure showing successful insertion and expression of TRV2 and TRV2-LOB18 in the genome of lilium.

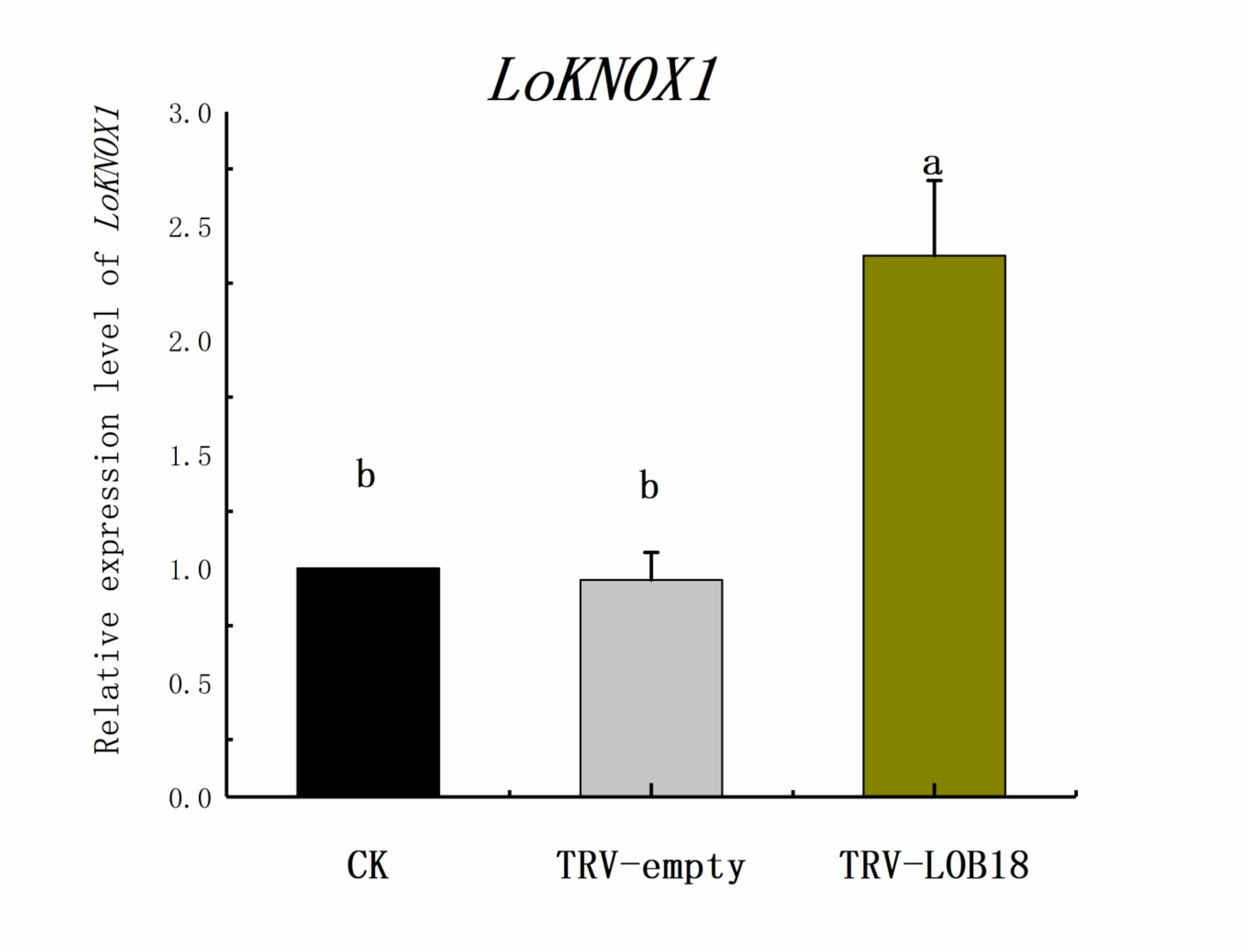

Figure S2. Relative expression of *LoKNOX1* after successful insertion of TRV2 and TRV2-LOB18. Lowercase letters indicate significance among treatment groups according to Duncan's multiple range test at $p < 0.05$.

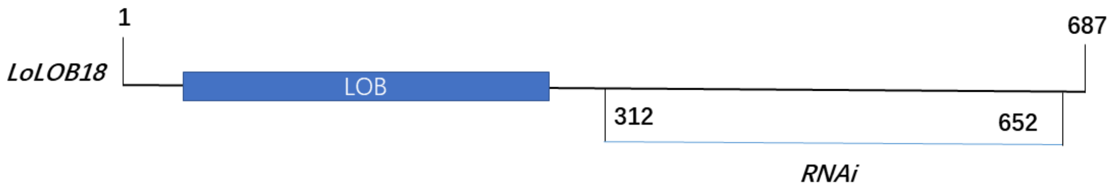

Figure S3. A model showing restriction sites during VIGS experiment.
